# The SMH approach as support for decision-making in a technical context

**DOI:** 10.3389/fnbeh.2022.942561

**Published:** 2022-10-18

**Authors:** Philipp Kranabitl, Clemens Faustmann

**Affiliations:** Institute of Machine Components and Methods of Development, Graz University of Technology, Graz, Austria

**Keywords:** decision making, bias, decision traps, model based development approach, systems thinking (ST), method

## Introduction

In society there is this widely common assumption, that engineers when developing a mechatronic product only make quantifiable and rational decisions. There are many details which lead to a successful product development, but the most important factor considering all aspects is sound decision-making. Eriksson even suggested, that product development can be viewed as a decision production system (Eriksson, 2009). Based on many failed engineering projects, the assumption that engineers mostly make rational decisions must be questioned. How to support decision-making is therefore a central topic in product development. In this paper the so called SMH Approach (S: system-thinking, M: model based, H: human factor) (Kranabitl et al., 2021) is presented. This short paper does not claim to address all relevant aspects of this far-reaching Research Topic in detail but aims to give an overview of the core steps which improve the decision-making capabilities of engineers based on a methodological concept in order to maximize the chances of success for product development. To better understand the human factor, creditions are integrated as a so far neglected human capacity which is rooted in brain function (Angel and Seitz, 2016).

In literature, many approaches exist, that have the purpose to support decision-makers. Many of them are summarized under the term “decision support systems,” defined by Little as model-based set of procedures for processing data and judgments to assist a manager in his decision-making (Little, 2004).

When developing a powertrain, a usual method which is applied after the design phase is the FMEA (failure mode and effect analysis). In this method, engineers from different disciplines (mechanical, electric/electronics, and software) work together to define and assess possible weak spots of the design. Engineers have to define possible failures and rate them on a scale from one to ten in three categories (severity, occurrence, and detection). This is considered one of the key methods in product development since design changes and expensive verification activities are based on the outcome of this method. The comparison of powertrain projects showed, that engineers made different assessment to similar failure modes on different occasions. Therefore, to propose a concept for decision support, one has to consider human aspects of decision-making processes. Kahneman (2003) distinguishes between two thinking systems. First, he describes system 1 that summarizes intuitive thinking which is fast, automatic, effortless, emotional and implicit. Secondly, he describes system 2 thinking, that is seen as slower but conscious, effortful, explicit, and logical way of thinking. In an ideal approach the decision-maker should rely on system 2 thinking, while considering that system 1 thinking happens unconsciously and can only be influenced by knowing the circumstances of the decision, such as the situation, the emotions regarding the subjects etc.

An approach to support decisions therefore has to consider the following aspects:

- Structured illustration of the decision context and possible affected aspects.
- Traceability of the information that is the base for the decision to be made.
- Emphasize the impact of the human factor on the decision output.

The developed decision-support concept, the so-called SMH approach (Kranabitl et al., 2021), considers these aspects and reflects them in its methodical steps.

## The SMH approach

The situation for companies in the automotive domain is defined by vast competition, cost pressure, legislative regulations, the emergence of new technologies, and changing customer expectations. In consequence, the future of companies in this industry heavily depends on making the right decisions in both a technical and strategic sense. To deepen the understanding of the presented theory in this paper, an example will be discussed with every step of the SMH approach. On what powertrain variant a company should focus on and consequently invest R&D budget over the next years to maintain profitable on a very competitive market is discussed. A wrong decision on questions like this, could cost companies large amounts of market share and therefore have to be considered very thorough.

**Figure 1:**
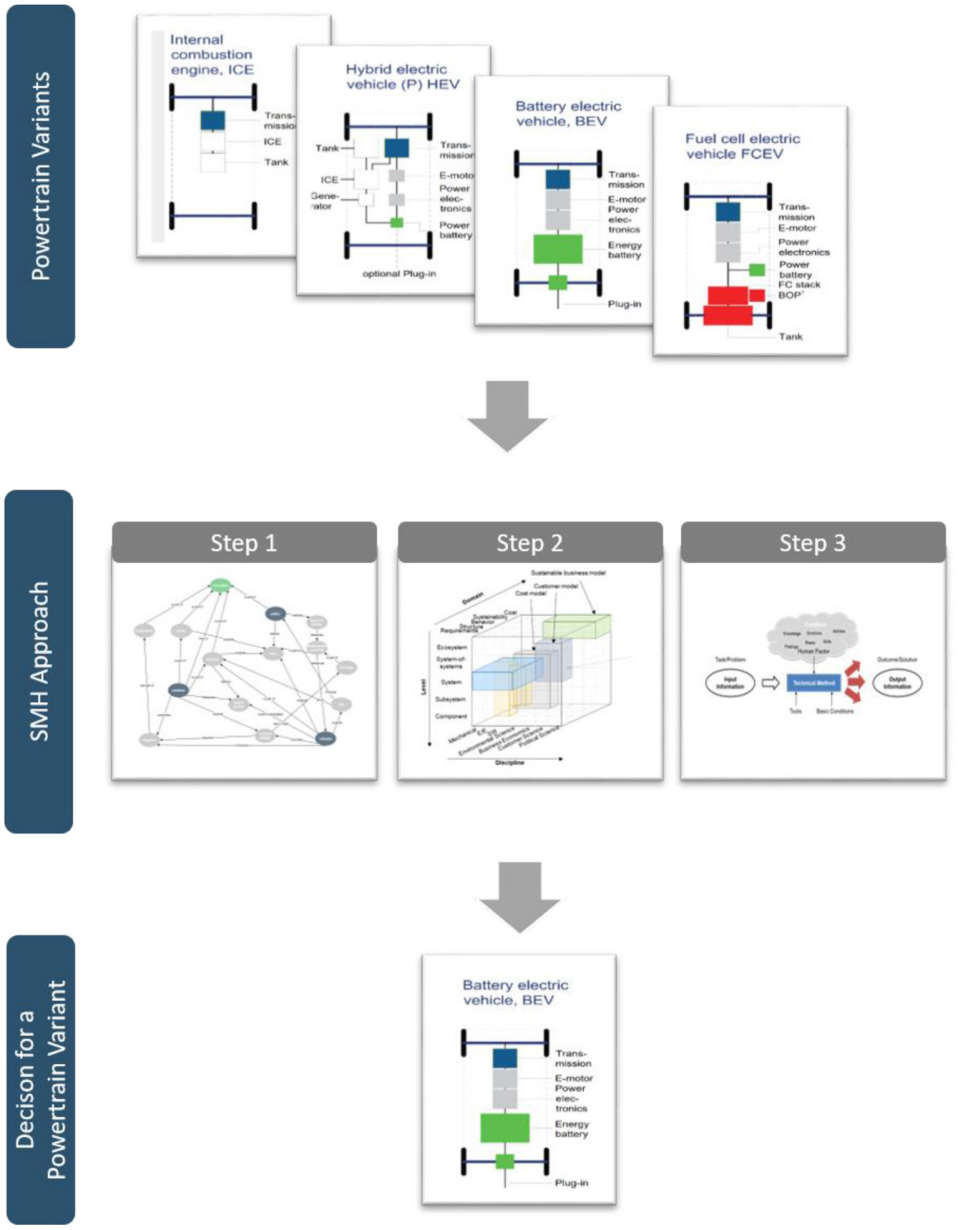
Generic illustration of the SMH approach to support decision-making in the context of powertrain development, inspired by Kranabitl et al. (2021).

Figure 1 shows the decision situation with options to choose from (powertrain variants), the three steps of the SMH approach (which are discussed in detail in the next subsections) and the option chosen based on the evaluation of the SMH approach.

### Systems thinking to define decision context

To define the context of a decision, including the situation it is made within, the stakeholders of the decision as well as the influences on society environment and more, systems thinking is necessary. As a key principle of systems engineering, systems thinking is a philosophy and state of mind to think beyond the system under development (Haberfellner et al., 2015). The decision on which powertrain to invest R&D resources isn't only a technical question. Political decisions, customer perception, environmental situations, infrastructure development and many more aspect also play a role in whether the developed product will be a success on the market or not. Therefore, the aim of this first step of the SMH approach is to widen the view of the decision maker in order to consider all relevant aspects for this decision.

To visualize the results of this first step, a semantic network including various aspects that are linked to this decision is considered. A semantic network basically consists of nodes (objects) and links (relations) between them (Bajzek et al., 2021). This developed network can be reused and adapted in future projects and illustrates the considered aspects of possible implications of a decision.

### Information base in form of models

As described in the first step, a decision-making process takes several aspects into account. To describe these different aspects, many models are required. In this second step of the approach, these models are collected, adapted, generated. Models in this matter reach from a technical model like a structural or functional model of a subsystem of the powertrain to models which aim to represent the preferences of future customer or political situations. Of course, models of future customer or political situations can only be modeled with a high degree of uncertainty. Yet these models are of high importance. Models with high and known uncertainty (the decision-maker should be aware of the model accuracy) should be embedded into the model landscape and updated when necessary instead of being not considered due to lack of significance. If a model with high uncertainty turns out to be wrong after some time, it needs to be updated and the changes to the whole decision situation have to be evaluated.

In the SMH approach all models are structured with the concept of a three-dimensional model cube to maintain an overview. This cube represents the sum of the considered models and structures these models in three dimensions: discipline, technical domain and level (Hick et al., 2019).

### Consideration of the human factor

Influences on decision can be understood by considering the way human brains work (Hammond et al., 2015). In literature, some of these influences are summarized in decision traps and biases by scientists such as Hammond et al. (2015), Korhonen and Wallenius (2020), or Kahneman et al. (2021).

The SMH approach includes human influences on a decision by forcing its applicant to consider decision traps and credition aspects (Hick et al., 2021). While the human influences are not yet quantified in the SMH approach, it provides a framework for decision-makers to consider the anchoring trap, status-quo trap, sunk cost trap, confirming evidence trap, simplicity trap and credition aspects in a structured way. As discussed in step two, models which have a low level of accuracy due to high uncertainty need to be interpreted as such by the decision maker. Considering the confirming evidence bias for this matter, a model which is of low accuracy but confirms the decision-makers initial believe, may be not considered as the vague information it represents but as way more accurate. Misjudgments due to flaws in human judgement like these are a threat to good decision-making and need to be reduced by following a proven process. This process can consist of a few simple questions which force the decision-maker to question if the derived belief is influenced by one of those biases. It can also dictate different people who have to confirm those beliefs in order to continue. The C-E-C (cognitions, emotions, creditions) triangle by Angel which states that forming a belief or believing is not possible without an associated emotion and cognition, forms the basis for the considered aspects in this step (Angel, 2017).

## Discussion

This short article does not aim to explain all steps of the SMH approach in detail but give an overview of core tasks and objectives. Further research is required to develop concepts and eventually applicable methods for persons in charge that have to make decisions. The chosen example of a powertrain variant selection illustrates that such a decision has many possible implications on the company, the society and the environmental system. A decision that appears to be solely of technical nature at first sight, such as the described example, has huge impact on the company's future economic success which leads to investment in research for new technologies or more employees. This directly influences society by providing work for people and by taxes as a result of sales and profits. Furthermore, the decision for a technology affects the environment because of required resources, CO_2_ equivalent emissions, and many more aspects.

The SMH approach is a concept to support responsible persons in taking decisions with a technical context. As this approach is more described on sja theoretical basis, future work regarding its implementation has to make sure that the following prerequisite are considered and fulfilled:

- Quantified parameters based on the models and the semantic network to better illustrate the possible options.
- Implementation in form of a method that is simplified to a certain extent, to ensure quick application also for decision-makers that are non-researchers in decision-making theory.
- Providing traceability of taken decisions and the information they are based on.

The SMH approach describes steps to extensively prepare the base or input for a decision by analyzing the situation, by relying on models as main source of information, and by considering the human factors in decision-making. It does not describe how the ideal trade-off between several factors is identified, such as in classical multicriteria decision models. The decision-maker is still challenged to draw conclusions out of the developed decision input in form of the semantic network and models. In the future this approach has to be enhanced and implemented as an IT solution to provide benefits for decision-makers in the sense of an expert system (Butler et al., 1997).

A further interesting addition to the proposed approach is to consider uncertainties in a quantified way and to apply statistics (Pfeifer and Lüthi, 1987). Especially for models which describe a future aspect, such as a model that describes the customer behavior when using the vehicle, a degree of uncertainty has to be considered. E.g., the customer might use the car in 10 years as a shared vehicle with other people rather than using it alone for weekend trips.

## Author contributions

CF was in a key role for bringing the core idea to paper. All authors contributed to the article and approved the submitted version.

## Funding

CF and PK declare that this study received funding from Siemens Healthineers. The funder was not involved in the study design, collection, analysis, interpretation of data, the writing of this article, or the decision to submit it for publication.

## Conflict of interest

The authors declare that the research was conducted in the absence of any commercial or financial relationships that could be construed as a potential conflict of interest.

## Publisher's note

All claims expressed in this article are solely those of the authors and do not necessarily represent those of their affiliated organizations, or those of the publisher, the editors and the reviewers. Any product that may be evaluated in this article, or claim that may be made by its manufacturer, is not guaranteed or endorsed by the publisher.
